# Unravelling exceptional acetylene and carbon dioxide adsorption within a tetra-amide functionalized metal-organic framework

**DOI:** 10.1038/ncomms14085

**Published:** 2017-02-08

**Authors:** Florian Moreau, Ivan da Silva, Nada H. Al Smail, Timothy L. Easun, Mathew Savage, Harry G. W. Godfrey, Stewart F. Parker, Pascal Manuel, Sihai Yang, Martin Schröder

**Affiliations:** 1 School of Chemistry, University of Manchester, Oxford Road, Manchester M13 9PL, UK; 2 ISIS Neutron Facility, STFC Rutherford Appleton Laboratory, Chilton, Oxfordshire OX11 0QX, UK; 3 School of Chemistry, University of Nottingham, Nottingham NG7 2RD, UK; 4 School of Chemistry, Cardiff University, Cardiff CF10 3XQ, UK

## Abstract

Understanding the mechanism of gas-sorbent interactions is of fundamental importance for the design of improved gas storage materials. Here we report the binding domains of carbon dioxide and acetylene in a tetra-amide functionalized metal-organic framework, MFM-188, at crystallographic resolution. Although exhibiting moderate porosity, desolvated MFM-188a exhibits exceptionally high carbon dioxide and acetylene adsorption uptakes with the latter (232 cm^3^ g^−1^ at 295 K and 1 bar) being the highest value observed for porous solids under these conditions to the best of our knowledge. Neutron diffraction and inelastic neutron scattering studies enable the direct observation of the role of amide groups in substrate binding, representing an example of probing gas-amide binding interactions by such experiments. This study reveals that the combination of polyamide groups, open metal sites, appropriate pore geometry and cooperative binding between guest molecules is responsible for the high uptakes of acetylene and carbon dioxide in MFM-188a.

## Abstract (web summary)

Understanding the mechanism of gas-sorbent interactions at a molecular level is important for the design of improved gas storage materials. Here, the authors study the binding domains of carbon dioxide and acetylene in a tetra-amide functionalized metal-organic framework at crystallographic resolution.

The emergence of metal-organic frameworks (MOFs) as multifunctional materials results from the combination of their high porosity and precise design of pore functionality. This allows fine tuning of pore size and geometry, framework topology and chemical functionality for targeted applications such as gas storage, separation, catalysis, sensing and proton conductivity^1^,^2^,^3^,^4^,^5^,^6^. Gaining in-depth understanding and control of the supramolecular host–guest interactions is crucial if practical materials are to be developed^7^. Two strategies have been commonly reported to enhance the host–guest interactions in MOFs: (i) incorporation of open metal sites (OMSs); (ii) functionalization of the organic linkers to provide binding sites for guest species. The favourable role of OMSs in guest binding has been widely confirmed. For example, *in situ* neutron diffraction experiments for D_2_-loaded NOTT-101 (ref. 8) and NOTT-112 (ref. 9), CO_2_-loaded MOF-74Mg (ref. 10), C_2_D_2_-loaded HKUST-1 (ref. 11) and MOF-74Fe (ref. 12), CD_4_-loaded MOF-74Mg (ref. 13) and D_2_, N_2_ and O_2_-loaded Cr-BTT^14^ (BTT^3−^==1,3,5-benzenetristetrazolate) clearly conclude that OMSs are the primary binding domains for guest molecules. This observation has been further evidenced by neutron spectroscopic experiments, which confirmed the large translational and/or rotational hindrance of bound guest molecules at OMSs^15^. In contrast, experimental investigations of the role of organic functionalization in guest binding in MOFs are scarce^16^,^17^,^18^, with the vast majority of reported examples relying predominantly on computational modelling^19^,^20^,^21^.

The amide group (-OCNH-) is of particular interest because it offers dual-functionality from both CO- and NH- sites. Notably, the introduction of amide groups in MOFs has been reported to strengthen the host–guest interactions in MOFs^19^,^22^,^23^. However, experimental observation of the precise role of amides in guest binding in MOFs is still largely lacking. Here we report the synthesis, crystal structure and gas uptake properties of the tetra-amide tetra-isophthalate Cu^II^ complex MFM-188 (MFM, Manchester Framework Material), which exhibits an exceptionally high CO_2_ uptake (120 cm^3^  g^−1^ at 298 K and 1.0 bar), and significantly, a record high C_2_H_2_ uptake (232 cm^3^ g^−1^ at 295 K and 1.0 bar) among porous MOFs under the same conditions. Gas binding is investigated by a combination of neutron powder diffraction (NPD) and inelastic neutron scattering (INS) for both CO_2_- and C_2_H_2_-loaded MFM-188. These crystallographic and dynamic experiments successfully establish a detailed molecular mechanism for guest binding at a crystallographic resolution.

## Results

### Synthesis and structural determination of MFM-188

**Figure 1:**
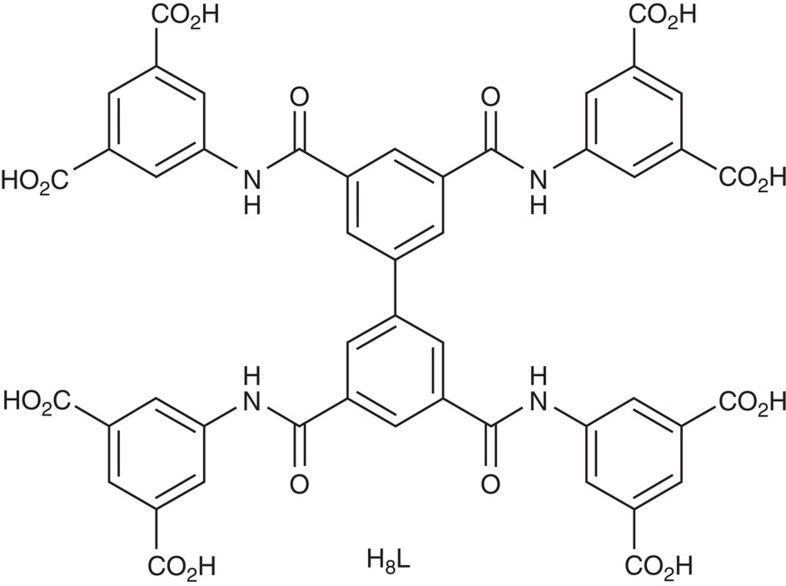
The tetra-amide octacarboxylate linker. Chemical structure of the tetra-amide octacarboxylate linker used in the synthesis of MFM-188.

The ligand H_8_L (Fig. 1), 5,5′,5′′,5′′′-([1,1′-biphenyl]-3,3′,5,5′-tetracarbonyl)tetrakis(azanediyl)tetraisophthalic acid, has been designed and synthesized (Supplementary Fig. 1). Single crystals of MFM-188 were obtained by solvothermal reaction of CuCl_2_ and H_8_L (ratio=5:1) in a acidic (HCl) mixture of dimethyl sulfoxide/*N*,*N*-diethylformamide/ethanol/H_2_O (v:v:v:v=1:2:1:1) at 80 °C for 36 h. MFM-188 was isolated as uniform blue square-plate crystals (size of 0.14 × 0.14 × 0.01 mm) in a yield of 64%. MFM-188 crystallizes in the space group *P*4/*mnc* [*a*=18.6861(7) Å; *c*=34.6960(18)Å]. The dihedral angle between each isophthalate and the main plane of the biphenyl core is 75.3° (Supplementary Fig. 1b), with each L^8-^ ligand linking to eight {Cu_2_} paddle-wheels, which each connect to four independent ligands to afford a three-dimensional (3D) open framework [Cu_4_(OH_2_)_4_L] (Supplementary Data 1). In terms of topology, the ligand can be viewed as an ensemble of three-connected (3-c) nodes: two for the biphenyl core and four corresponding to the isophthalate moieties, with the {Cu_2_} paddle-wheels serving as four-connected (4-c) nodes. Thus, the underlying trinodal (3,3,4-c) net with stoichiometry (3-c)_2_(3-c)(4-c) presents a rare example of the ***lwg*** topology (Supplementary Fig. 2)^24^.

**Figure 2:**
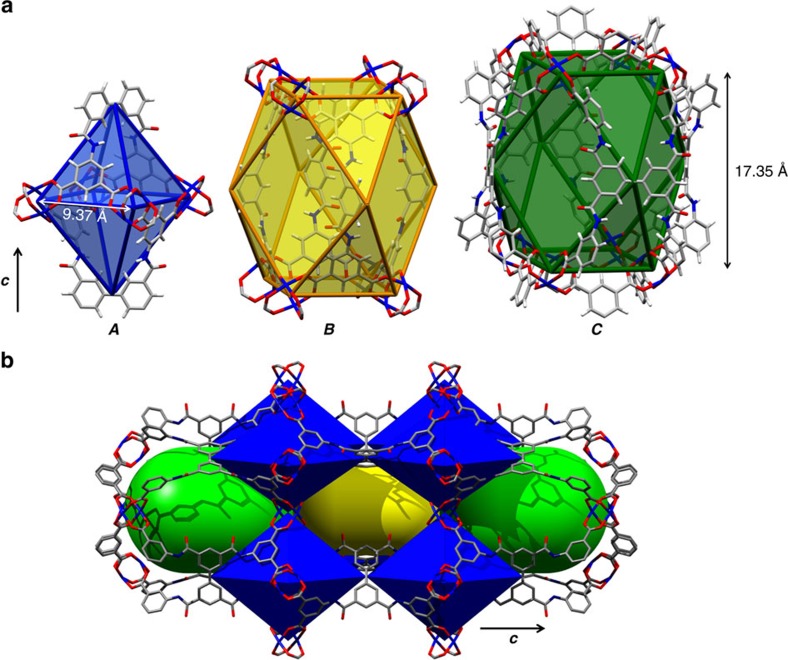
View of the crystal structure of MFM-188. (**a**) Polyhedral representation of the three types of cages: ***A*** (octahedral), ***B*** and ***C*** (cuboctahedral). (**b**) The assembly of these cages in 3D space forming the MFM-188 framework. Cages ***A***,***B*** and ***C*** are highlighted in blue, yellow and green, respectively.

The structure of MFM-188 can be viewed as 3D alternate packing of three types of metal-ligand cages (denoted as ***A***, ***B*** and ***C***) (Fig. 2a). The smallest cage ***A*** is comprised of four {Cu_2_} paddle-wheels and two ligands, and has an elongated, distorted octahedral geometry (internal size of ∼ 9.4 × 9.4 × 13.4 Å). The {Cu_2_} paddle-wheels occupy the four equatorial vertices with two biphenyl cores from the ligands occupying the apical vertices. Corner-sharing of cages ***A*** with biphenyl groups running along the *c* axis and with paddle-wheels along the *a/b* axis extends the structure (Fig. 2b). Two further distorted cuboctahedral cages (***B*** and ***C***) result from this assembly, each comprising of eight {Cu_2_} paddle-wheels and four ligands. The length of cage ***C***, defined by the separation between the centroids of the two closest {Cu_2_} paddle-wheels along the *c* direction, is 17.4 Å. Each Cu^II^ site has a coordinated water molecule at the axial position and points to the centre of Cage ***C***. Cages ***B*** and ***C*** are connected through shared lozenge-shaped windows on the *ab* plane and through their apical square-shaped windows along the *c* direction. Taking the van der Waals radii into account, cages ***B*** and ***C*** have internal pore size of ∼ 11 × 11 × 17 Å and ∼ 17 × 17 × 17 Å, respectively. Each cage ***B*** and ***C*** is surrounded by eight cages ***A***, while each cage ***A*** is surrounded by four cages ***B*** and four cages ***C***, with cages ***A***, ***B*** and ***C*** present in a 2:1:1 ratio. The total accessible volume of MFM-188 upon removal of guest solvents is 73% using the PLATON/VOID routine^25^. Within the void space of MFM-188, there is a high concentration of amide (-OCNH-) groups pointing into cage ***B*** (O-centres) and into cage ***C*** (N-H centres), thus endowing these cages with a combination of open metal sites and multiple functional groups. In contrast, none of the amide groups protrude into cage ***A***, which is functionalized solely with phenyl rings.

### Characterization of MFM-188

The purity of bulk MFM-188 material was confirmed by powder X-ray diffraction (Supplementary Fig. 3) and elemental analysis. Activated MFM-188a was prepared by solvent exchange with methanol and then flowing supercritical CO_2_ through the sample for 12 h followed by heating at 100 °C under dynamic vacuum for 20 h. N_2_ adsorption at 77 K shows a reversible type I isotherm (Supplementary Fig. 4) for MFM-188 with a Brunauer Emmett Teller surface area of 2568, m^2^ g^−1^ (Supplementary Fig. 5). The pore size distribution, calculated using a non-local density functional theory model, reveal two broad peaks centred at 12.5 and 14.7 Å, in good agreement with the pore size measured for cages ***A***, ***B*** and ***C***. Importantly, the total pore volume measured from the N_2_ isotherm (1.12 cm^3^ g^−1^) compares favourably with that (1.07 cm^3^ g^−1^) calculated from the X-ray crystal structure, confirming complete activation of the material. The overall porosity of MFM-188a is moderate in comparison with the most porous MOFs reported to date^26^.

### CO_2_ and C_2_H_2_ adsorption analysis

**Figure 3:**
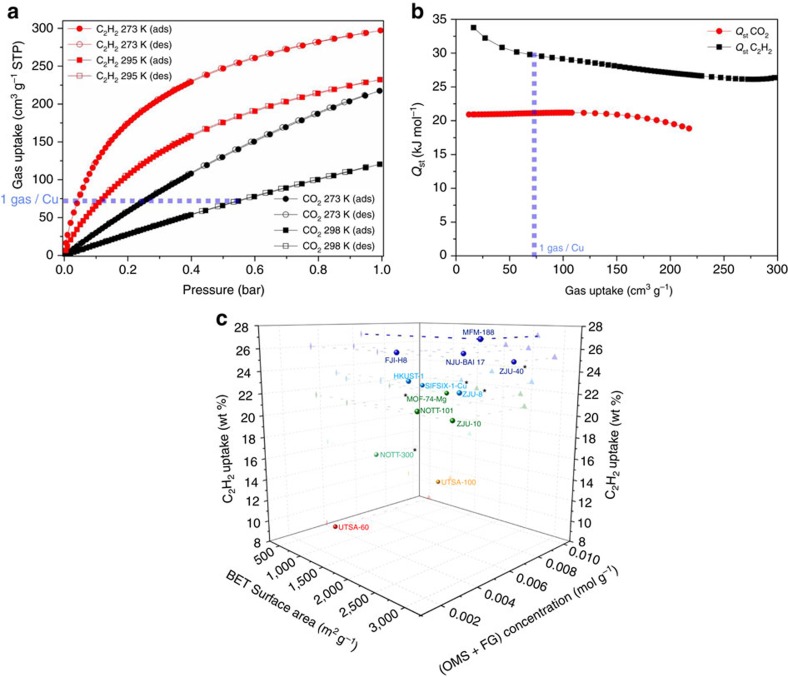
Gas adsorption isotherms for MFM-188a. (**a**) Comparison of the CO_2_ and C_2_H_2_ adsorption isotherms for MFM-188a at 273-298 K and 1.0 bar. (**b**) Variation of the isosteric heats of adsorption for CO_2_ and C_2_H_2_ adsorption in MFM-188a. (**c**) 3D plot of 295 K C_2_H_2_ uptake as a function of BET surface area and total gravimetric concentration of open metal sites and functional groups for selected MOFs. For clarity, only the best-behaving and representative MOFs are shown in the plot (* data were obtained at 298 K).

The uptake of CO_2_ and C_2_H_2_ by activated MFM-188a was investigated up to 1 bar, and in both cases the isotherms show fully reversible adsorption with a CO_2_ uptake of 120 cm^3^ g^−1^ (23.7 wt% or 86.7 v/v) recorded at 298 K and a C_2_H_2_ uptake of 232 cm^3^ g^−1^ (27.0 wt% or 166.7 v/v) at 295 K (Fig. 3a). Importantly, these gravimetric uptakes are amongst the highest reported (Table 1), and are only outperformed in the case of CO_2_ adsorption by MOF-74Co (30.6 wt%) and MOF-74Mg (35.2 wt%)^27^, both of which present a narrower pore channel and a much higher density of OMSs (MOF-74Co: 6.4 mmol g^−1^; MOF-74Mg: 8.2 mmol g^−1^; MFM-188: 3.3 mmol g^−1^). Interestingly, MFM-188a shows the highest CO_2_ uptake of other amide-functionalized MOFs such as the ***rht***-[Cu_3_(TPBTM)]^22^ (23.3 wt%) and ***fof***-NOTT-125 (ref. 19) (18.2 wt%). The CO_2_ uptake of MFM-188a at 0.15 bar is 3.9 wt%. To the best of our knowledge, the gravimetric capacity of C_2_H_2_ (27.0 wt%) in MFM-188a represents the highest value observed to date for porous solids, exceeding FJI-H8 (ref. 11 (26.0 wt%)), NJU-Bai-17 (ref. 23 (25.8 wt%)), HKUST-1 (ref. 28 (23.3 wt%)) and SIFSIX-1-Cu^29^ (22.1 wt%). The CO_2_ and C_2_H_2_ uptake capacity in MFM-188a increase considerably at 273 K to 217 cm^3^ g^−1^ (42.9 wt%) and 297 cm^3^ g^−1^ (34.5 wt%), respectively.

The *Q*_st_ values at zero coverage determined by virial analysis of adsorption isotherms (Supplementary Figs 6 and 7) are moderate but comparable to typical Cu^II^ MOFs where adsorption of gas molecules occurs primarily on OMSs^21^,^22^,^23^,^28^,^30^, with *Q*_st_ (CO_2_)=21.0 kJ mol^−1^ and *Q*_st_ (C_2_H_2_)=32.5 kJ mol^−1^. Interestingly, the *Q*_st_ plots do not display any major decrease across the whole adsorption range (Fig. 3b), suggesting the presence of other favourable binding sites in addition to OMSs. We were thus motivated to investigate further the role of amides and OMSs in CO_2_ and CH_2_ adsorption in MFM-188a.

### Determination of adsorption domains for CO_2_ and C_2_H_2_

**Figure 4:**
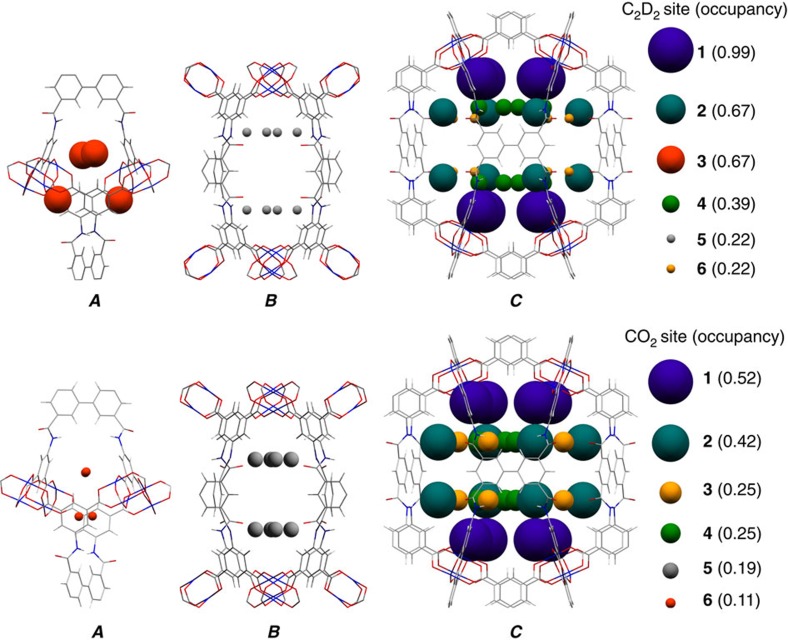
Distribution of adsorbed gas molecules within three types of cages in MFM-188a determined from NPD data. Representation of C_2_D_2_ (top) and CO_2_ (bottom) positions in the cages ***A***,***B*** and ***C*** of MFM-188a at a loading of 1.75 CO_2_/Cu and 3.2 C_2_D_2_/Cu, respectively. The radii of the coloured balls figuring the various sites are proportional to corresponding crystallographic occupancies.

The locations of adsorbed CO_2_ and C_2_D_2_ molecules within desolvated MFM-188a were determined by *in situ* NPD as a function of gas loading. NPD patterns were recorded at 7 K for the desolvated material (Supplementary Fig. 8) and at loadings of 1.75 CO_2_/Cu (127 cm^3^ g^−1^) (Supplementary Fig. 9) and 3.2 C_2_D_2_/Cu (234 cm^3^ g^−1^) (Supplementary Fig. 10), which correspond to the adsorption uptakes at 1 bar and room temperature for the respective gas species. Fourier difference map analysis of the NPD data of the desolvated MFM-188a indicates no residual nuclear density peak in the pore, thus confirming the complete activation and structural integrity of the desolvated sample (Supplementary Data 2). Loading of CO_2_ or C_2_D_2_ in MFM-188a was accompanied by significant changes in the relative peak intensities of the diffraction patterns. Successive Fourier difference map analysis followed by Rietveld refinement allowed identification of the position, occupancy and orientation for adsorbed gas molecules within the framework cages (Supplementary Data 3 and 4). In both cases, six different sites (**1**, **2**, **3**, **4**, **5** and **6** in the order of decreasing occupancy) were observed for adsorbed guest molecules: one in cage ***A***, one in cage ***B*** and four in cage ***C***, all of them having the same crystallographic multiplicity (Fig. 4). Notably, 83% of loaded CO_2_ and 72% of loaded C_2_D_2_ molecules were found in cage ***C***, which contains open Cu^II^ sites and the N-H sites of amides pointing to the centre of the cage. The smallest cage ***A*** plays a different role in CO_2_ and C_2_D_2_ uptakes with a minimum contribution to the CO_2_ adsorption, but considerable effect to the C_2_D_2_ binding.

**Figure 5:**
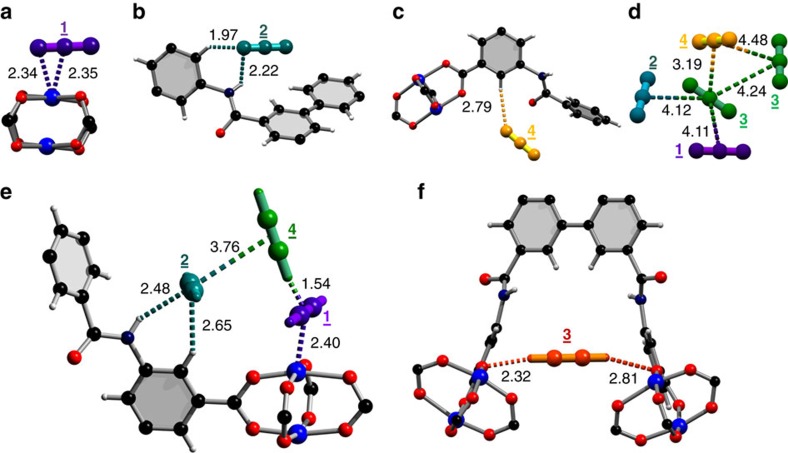
Crystallographic resolution of CO_2_ and C_2_H_2_ binding in MFM-188a. Framework atoms: C, black; O, red; H, white; N, blue; Cu, blue. Extra framework gas molecules are slightly magnified and coloured according with the binding site they occupy. Distances are shown in Å. View of the binding of adsorbed CO_2_ molecules at site **1** (**a**), **2** (**b**) and **4** (**c**). (**d**) View of the packing of adsorbed CO_2_ molecules within cage ***C***. View of the cooperative binding of adsorbed C_2_H_2_ molecules at site **1**, **2, 4** (**e**) and at site **3** (**f**).

In the structure of MFM-188·7CO_2_, the CO_2_ molecules at site **1** bind at OMSs in cage ***C*** (occupancy=0.52). The linear body of CO_2_(**1**) is perpendicular to the Cu–Cu axis with C_CO_2__···Cu=2.35(3), O_CO_2__···Cu=2.34(3) Å (Fig. 5a). The second most populated site **2** (occupancy=0.42) is also located in cage ***C*** where adsorbed CO_2_ molecules form H-bonds with both the free amide groups (-NH) and the adjacent isophthalate -CH groups [N···O_CO_2__=2.66(6) Å, <N-H···O=109°; C···O_CO_2__= 2.75(4) Å, <C-H···O=141°] (Fig. 5b). CO_2_(**3**) and CO_2_(**4**) are also found in cage ***C*** (occupancy=0.25 for both) at the centre of the cage and forming weak supramolecular interaction to isophthalate -CH groups [C···O_CO_2__=3.66(6) Å, <C-H···O=156°] (Fig. 5c,d). The remaining CO_2_(**5**) and CO_2_(**6**) sites are found in cage ***B*** and ***A*** (occupancy=0.19 and 0.11, respectively). Given the long distances between CO_2_(**5**)/CO_2_(**6**) and the framework surface (above 5 Å), no specific binding interaction could be identified, thus excluding the presence of dipole interactions between the amide –C=O and adsorbed CO_2_ molecules in cage ***B***.

MFM-188·12.8C_2_D_2_ exhibits a different distribution of adsorbed C_2_D_2_ molecules within the cages, indicating the presence of different gas-sorbent binding mechanisms. The OMSs are fully occupied by C_2_D_2_(**1**) molecules with a side-on interaction between the C≡C bond and the Cu^II^ centre, Cu···C=2.60(3), 2.37(3)Å (Fig. 5e), similar to those determined for C_2_D_2_-loaded HKUST-1 (ref. 28). C_2_D_2_(**2**) and C_2_D_2_(**3**) are found in cage ***C*** and ***A***, respectively, (same site occupancy of 0.67). C_2_D_2_(**2**) forms H-bonds via its C≡C(δ-) π electrons to the N-H groups pointing into the pore [N···C_C2H2_=3.24(5) Å, <N-H···C=147^o^] and electrostatic supramolecular interactions with the C-H groups from the adjacent isophthalate group [C···C_C2H2_=3.47(1) Å, <C-H···C=149^o^]. Significantly, this represents the first example of formation of H-bonding between the C_2_H_2_ and an amide group in the solid state. C_2_D_2_(**3**) is located at the triangular window of the smallest octahedral cage ***A*** where C_2_D_2_ molecules are H-bonded to carboxylate oxygen atoms [C_C2H2_···O=3.72(4), 3.37(4) Å, <C-D···O=143°, 169^o^] (Fig. 5f). In addition, the combination of C_2_D_2_(**1**) and C_2_D_2_(**2**) generates another adsorption site (**4**) with an occupancy of 0.49 in cage ***C***, stabilized via the intermolecular C_2_D_2_···C_2_D_2_ dipole interactions (Fig. 5e). Specifically, C_2_D_2_(**4**) is perpendicular to C_2_D_2_(**1**) and strong intermolecular H-bonding is observed between these two sites [C^**4**^···C^**1**^=2.58(5)Å, <C-D^**4**^···C^**1**^=164°]. C_2_D_2_(**4**) is reinforced by C_2_D_2_(**2**) through π···π interactions of 3.76(1)Å and a dihedral angle of 84°. C_2_D_2_(**5**) and C_2_D_2_(**6**) are found in cage ***B*** and ***C***, respectively, (occupancy of 0.22 for both) and without specific binding interaction to the MOF host. Thus, C_2_D_2_ displays a dual role behaving as an H-acceptor from amides N-H and aromatic C-H groups, but a H-donor to the carboxylate oxygen, thus enhancing the C_2_D_2_-MOF-binding strength. In contrast, CO_2_ participates in H-bond formation as an acceptor only. The highly cooperative binding of acetylene at both the open Cu^II^ sites and free amides, coupled with the well-defined micropore windows, leads to the record high C_2_H_2_ adsorption capacity in MFM-188a.

### Inelastic neutron scattering of gas-sorbent dynamics

**Figure 6:**
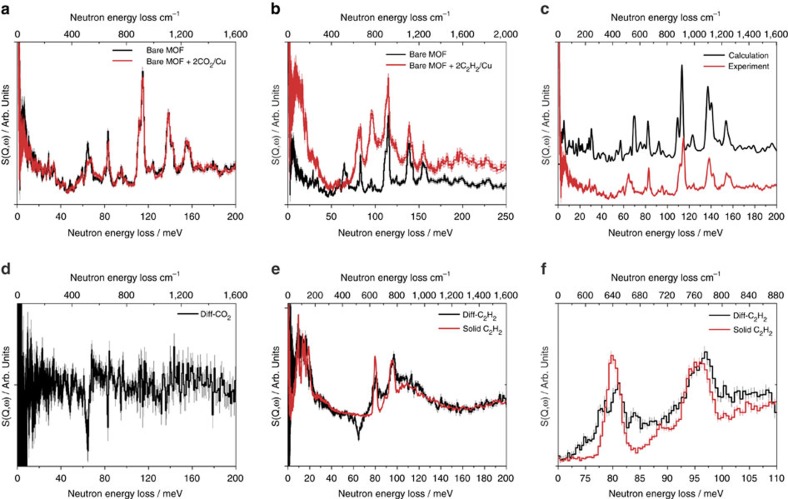
INS spectra for MFM-188a as a function of guest loading. Comparison of the INS spectra for bare MFM-188a and for (**a**) CO_2_ and (**b**) C_2_H_2_-loaded MFM-188a. (**c**) Comparison of the experimental and DFT calculated INS spectra for MFM-188a. Comparison of the difference plots for INS spectra of bare MFM-188a and the (**d**) CO_2_ and (**e**) C_2_H_2_-loaded MFM-188a, and the experimental INS spectra of condensed (**e**) C_2_H_2_ in the solid state. (**f**) Enlarged details for the INS spectra showing the C_2_H_2_ internal vibrational modes.

Static crystallographic experiments are unable to gain dynamic insight into the gas-sorbent systems. To directly visualize the binding dynamics of adsorbed CO_2_ and C_2_H_2_ molecules with accessible functional groups, INS was measured for MFM-188a as a function of CO_2_ and C_2_H_2_ loading at 11 K (Fig. 6). The INS spectra for the bare MOF show multiple features, fully modelled via DFT calculations (Fig. 6c). The peak at 65 meV corresponds to the out-of-plane wagging modes of the N-H group, and peaks at 84 and 112-156 meV originate from various deformational motions of the phenyl rings and wagging modes of aromatic C-H groups. Comparison of the INS spectra for bare and CO_2_-loaded MFM-188a shows an overall stiffening effect as evidenced by a global shift of peaks to slightly higher energy (Fig. 6a). In addition, a noticeable change in the peak at 65 meV and several small changes to the peaks between 84 and 156 meV were observed, indicating the reduction of the motion of both amide N-H and aromatic C-H groups (Fig. 6d). This suggests the formation of H-bonds between CO_2_ molecules and these functional groups, particularly with amide N-H sites.

In comparison, the INS spectra of C_2_H_2_-loaded MFM-188a shows significant increase in intensity due to the recoil motion from the H atoms on adsorbed C_2_H_2_ (Fig. 6b). Comparison of the difference INS spectra (i.e., signals from adsorbed C_2_H_2_ and changes of the local MOF modes) and that of the solid C_2_H_2_ shows a number of interesting observations. Firstly, the low-energy INS peaks (below 25 meV, assigned as the translational modes of C_2_H_2_ molecules) of the difference spectra shift slightly to the lower energy region but maintain the resolved threefold peak profiles as observed in free solid C_2_H_2_. This indicates that the C_2_H_2_ molecules are ordered with restricted translational motions within MFM-188a. Secondly, the INS peaks at 80 and 95 meV (assigned as the acetylene asymmetric and symmetric C–H bending mode, respectively) split from a single-peak profile to a double-peak profile upon adsorption, indicating the presence of adsorbed species resulting from slightly different binding energies to the MOF host (Fig. 6f). This is in excellent agreement with the presence of the strongly bound C_2_H_2_ molecules to the OMSs and free amides, and weakly bound C_2_H_2_ molecules to the phenyl rings and in the centre of the cages, as observed by NPD. Thirdly and most importantly, the INS peak at 65 meV (assigned as the out-of-plane wagging modes of the N-H group) disappears completely upon inclusion of C_2_H_2_ molecules in the pore, indicating loss of this mode and confirming unambiguously the direct formation of strong H-bonds between the free amides (N-H) and C_2_H_2_ molecules, as found in the NPD model. This observation confirms the first example of H-bonding between an amide group (N-H) and C_2_H_2_ in the solid state. Overall, the INS study is in excellent agreement with NPD results and confirms the crucial role of the free amides in the pore for gas uptakes.

## Discussion

MFM-188a shows excellent performance for potential applications for C_2_H_2_ storage (27.0 wt%) and exhibits high adsorption uptake of CO_2_ (23.7 wt%) under ambient conditions. A 3D plot summarising the relationship between BET surface area, density of ‘OMSs+functional groups' in the structure, and uptake of C_2_H_2_ is shown in Fig. 3c for a number of the best MOFs reported to date. It is clear that to achieve high C_2_H_2_ uptake capacity, integration of high BET surface area (and porosity to a wider extent) and high density of binding sites in the pore structure are required. However, this is often inherently contradictory since high surface area/porosity will naturally dilute the binding sites in the pore, leading to an inevitable trade-off between these two factors. MFM-188a displays a suitably high BET surface area and high density of binding sites owing to its framework topology and pore geometry, and therefore, shows a record high C_2_H_2_ uptake.

Although it is widely believed that the amide groups in pores will actively participate in gas adsorption via H-bonding, no cogent experimental evidence has been reported to date. The present study represents a unique example of a comprehensive investigation of the gas-sorbent binding interaction in a tetra-amide functionalized MOF via a combination of neutron diffraction and spectroscopic techniques. These experiments offer key insights into the molecular details of this host–guest system from both crystallographic and dynamic perspectives. A highly cooperative binding mechanism of CO_2_ and C_2_H_2_ was found on the OMSs and free amide groups in the pores of MFM-188a, with one site enhancing binding at the other. This work not only offers a new porous material for high-capacity CO_2_ and C_2_H_2_ storage, but also, more importantly, facilitates the design and structural optimization for new porous materials with improved performance in gas adsorption.

## Methods

### Materials and equipment

All chemical reagents and gases were obtained from commercial sources and unless otherwise noted were used without further purification. ^1^H and ^13^C NMR spectra were measured on a Bruker DPX 300, AV400 or AV(III)500 spectrometers. Residual protonated species in the deuterated solvents were used as internal references. Mass spectrometry was performed on a Bruker MicroTOF with the sample dissolved in MeOH or CH_3_CN. Elemental analyses were measured on a CE-440 Elemental Analyzer provided by Departmental Analytical Services at the Universities of Nottingham and Manchester. TGA was performed under a flow of air with a heating rate of 5 °C min^−1^ using a Perkin Elmer Pyris 1 thermogravimetric analyser. Powder X-ray diffraction measurements were carried out at room temperature on a PANalytical X′Pert PRO diffractometer using Cu-Kα radiation (*λ*=1.5418 Å) at a scan speed of 0.02° s^−1^ and a step size of 0.02° in 2*θ*.

### Preparation of H_8_L

[1,1′-biphenyl]-3,3′,5,5′-tetracarboxylic acid (3.0 g, 9.1 mmol) was suspended in 200 ml of dry THF and thionyl chloride (10 ml, 137.8 mmol) was added dropwise under inert atmosphere. The resulting mixture was refluxed under inert atmosphere for 16 h. After cooling, the solvent and excess thionyl chloride were evaporated under reduced pressure affording [1,1′-biphenyl]-3,3′,5,5′-tetracarbonyl tetrachloride as an off white solid, which was dried under vacuum for 2h and dissolved in 200 ml of dry THF. The [1,1′-biphenyl]-3,3′,5,5′-tetracarbonyl tetrachloride solution was added by cannula to a solution of 5-aminoisophthalic acid (9.84 g, 54.3 mmol) in 250 ml of dry THF and trimethylamine (3.7 ml, 27.4 mmol) was added slowly. The resulting suspension was stirred under inert atmosphere for 2 h. Aqueous HCl (2M, 200 ml) was added and THF evaporated under reduced pressure. The resulting solid was isolated by filtration, washed successively with water and hot MeOH and dried under vacuum to afford H_8_L as an off-white powder (7.65 g, 85.5%). ^1^H NMR: (300 MHz, DMSO-d_6_) *δ*=12.98 (bs, 8H, COOH), 11.07 (bs, 4H, NH), 8.83 (bm, 14H, ArH), 8.33 (bs, 4H, ArH); ^13^C NMR: (126 MHz, CDCl_3_), *δ*= 167.0 (CO), 165.5 (CO), 140.2 (C), 140.0 (C), 136.1 (C), 132.2 (C), 130.1 (CH), 127.6 (CH), 125.7 (CH), 125.3 (CH); MS: (ESI): *m/z*=981.31 (M-H)^−^.

### Preparation of MFM-188

H_8_L (165 mg, 0.168 mmol) and CuCl_2_ (0.225 g, 1.675 mmol) were dissolved in dimethyl sulfoxide (8 ml). N,N′-diethylformamide (16 ml), EtOH (16 ml) and aqueous HCl (8 ml, 0.1 M) were added to the resulting solution, which was placed in a tightly capped 250 ml Duran pressure plus laboratory bottle. The solution was heated at 80 °C in an oven for 96 h, and a large amount of crystalline product precipitated. The blue crystal plates were isolated by filtration while the mother solution was still warm and washed sequentially with warm DMF and MeOH, and stored under MeOH (yield based on TGA of as synthesized material: 0.172 g, 64%, see Supplementary Fig. 11). After activation and rehydration, [Cu_4_L(H_2_O)_4_]·12H_2_O was obtained; elemental analysis: calculated/found: C, 39.90/40.16; H, 3.21/3.24; N, 3.88/3.79.

### Material activation procedure

Aquamarine crystals of MFM-188 were solvent-exchanged with MeOH for 3 days, replacing with fresh MeOH twice a day. The MeOH exchanged samples were first activated by flowing supercritical CO_2_ for 12 h, upon which the colour changed to purple, then transferred to a glovebox under N_2_. The scCO_2_ dried samples were then transferred onto the gas adsorption instruments under inert atmosphere and heated at 100 °C under dynamic vacuum for 20 h.

### Single crystal X-ray diffraction structure determination

A single crystal diffraction data set for MFM-188 was collected at 120 K using an Agilent GV1000 diffractometer (*λ*=1.54056 Å). Details of data collection and processing procedures are included in the CIF files. Structures were solved by direct methods using SHELXS^31^ and remaining atoms were localized from successive difference Fourier maps using SHELXL^32^. The hydrogen atoms from the linkers and the coordinated water molecules were placed geometrically and refined using a riding model. The refinement of the framework was performed by ignoring the contribution of the disordered solvent molecules. The region containing the disordered electron density was identified by considering the van der Waals radii of the atoms constituting the ordered framework. The contribution of this region to the total structure factor was calculated via a discrete Fourier transformation and subtracted in order to generate a new set of *hkl* reflections by means of the program SQUEEZE^33^. Acquisition and final refinement data are summarized in Supplementary Table 1.

### Gas sorption measurements

Volumetric N_2_, CO_2_ and C_2_H_4_ isotherms for pressures in the range 0–1 bar were determined using a Micromeritics 3-Flex apparatus. Ultra-high purity He (99.999%), for void volume determination, N_2_ (99.999%), CO_2_ (99.999%) were purchased from BOC and used as received. C_2_H_2_ gas was filtered through 4A molecular sieves and activated carbon to remove traces of acetone. Pore size distribution data for MFM-188a was determined by analysing the N_2_ isotherm at 77K using a non-local density functional theory based on a carbon model containing cylindrical pores as implemented in the 3-Flex software package.

The CO_2_ adsorption isotherms at 273 and 298 K as well the C_2_H_2_ isotherms at 273 and 295 K were fitted to the virial equation (1). *P* is the pressure expressed in bars, *N* is the amount expressed in mol g^−1^, *T* is the temperature in K, *a*_*i*_ and *b*_*j*_ are virial coefficients and *m, n* represent the number of coefficients. The values of the virial coefficients *a*_0_ to *a*_*m*_ were then used to calculate the isosteric heat of adsorption using equation (2). *Q*_st_ is the coverage dependent isosteric heat of adsorption and R is the universal gas constant.


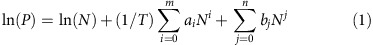



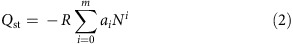


To evaluate the CO_2_/N_2_ selectivity of MFM-188a, the N_2_ isotherm was also measured at 298 K (Supplementary Fig. 12) up to 1 bar. By determining the ratios of the Henry's law constants from single-component isotherms, the CO_2_/N_2_ adsorption selectivity for MFM-188a is estimated as 22.5 at 298 K. It is worth noting that this selectivity carries large uncertainty because of the very low N_2_ uptake measured under these conditions.

### Neutron powder diffraction

NPD experiments were carried out at WISH, a long wavelength powder and single crystal neutron diffractometer at the ISIS Facility at the Rutherford Appleton Laboratory (UK)^34^. MFM-188 was loaded into a 6 mm diameter vanadium sample can and outgassed at 1 × 10^−7^ mbar and 100 °C for 1 day. The sample was loaded into a liquid helium cryostat and cooled to 7 K for data collection. CO_2_ and C_2_D_2_ gas was introduced by warming the samples to 290 K, and the gas dosed volumetrically from a calibrated volume. The gas-loaded sample was then cooled to 7 K over a period of 2 h to ensure good mobility of adsorbed CO_2_/C_2_D_2_ within the crystalline structure of MFM-188a. The sample was kept at 7 K for an additional 30 mins before data collection to ensure the thermal equilibrium. The structure solution was initially established by considering the structure of the bare MFM-188a, and the residual nuclear density maps were further developed from subsequent difference Fourier analysis using TOPAS program Acquisition and refinement data are summarized in Supplementary Table 2.

### Inelastic neutron scattering

INS spectra were recorded on the TOSCA spectrometer at the ISIS Facility at the Rutherford Appleton Laboratory (UK) for energy transfers between ∼−2 and 500 meV. In this region TOSCA has a resolution of 1.5% *ΔE/E*. Before INS experiments, MeOH exchanged MFM-188 was activated by flowing supercritical CO_2_ for 12 h, the desolvated sample was then transferred in a glovebox under inert atmosphere, and 0.5 g of activated MFM-188a were placed in a closed cylindrical vanadium sample cell with an indium seal. The sample was further degassed at 100 °C under dynamic vacuum for 20 h to remove traces of adsorbed guest species.

The temperature during data collection was controlled using a closed cycle refrigerator cryostat (11±0.1 K). The loading of 2C_2_H_2_/Cu and 2CO_2_/Cu was performed volumetrically at room temperature to ensure that C_2_H_2_ and CO_2_ were present in the gas phase when not adsorbed and also to ensure sufficient mobility of C_2_H_2_ and CO_2_ inside the MFM-188a framework. Subsequently, the temperature was reduced to 7 K for data collection. Background spectra (sample can plus MFM-188a) were subtracted to obtain the difference spectra. INS spectra for condensed C_2_H_2_ and CO_2_ in the solid state were measured by using a plate sample container. Approximately 1–2 l of each gas at room temperature was condensed slowly at temperatures below their melting points and cooled to temperature below 10 K for neutron scattering measurements.

### DFT calculations and modelling of the INS spectra

Periodic density functional theory (periodic-DFT) calculations were carried out using the plane-wave pseudopotential method as implemented in the CASTEP code^35^,^36^. Exchange and correlation were approximated using the PBE functional^37^. For MFM-188 the structure determined by NPD was used as the initial structure. The plane-wave cutoff energy was 750 eV using on-the-fly generated pseudopotentials and spin polarized calculations were carried out. Owing to the size of the unit cell (12106 Å^−3^), Brillouin zone sampling of electronic states was performed on 4 × 4 × 1 Monkhorst-Pack grid. The equilibrium structure, an essential prerequisite for lattice dynamics calculations was obtained by BFGS geometry optimization after which the residual forces were converged to zero within 0.007 eV Å^−1^. Phonon frequencies were obtained by diagonalization of dynamical matrices computed using density functional perturbation theory^38^. The atomic displacements in each mode that are part of the CASTEP output enable visualization of the modes to aid assignments and are also all that is required to generate the INS spectrum using the program ACLIMAX^39^. It was found that even with these stringent convergence conditions, there were five imaginary modes. It is emphasized that for all the calculated spectra shown the transition energies have not been scaled.

### Data availability

The X-ray crystallographic coordinates for structures reported in this Article have been deposited at the Cambridge Crystallographic Data Center (CCDC), under deposition numbers 1496263 (as-synthesized MFM-188), 1504308 (desolvated MFM-188a), 1496264 (C_2_D_2_-loaded MFM-188) and 1496265 (CO_2_-loaded MFM-188). These data can be obtained free of charge from The Cambridge Crystallographic Data Center via www.ccdc.cam.ac.uk/data_request/cif, all other data are available from the corresponding authors upon request.

## Additional information

**How to cite this article:** Moreau, F. *et al*. Unravelling exceptional acetylene and carbon dioxide adsorption within a tetra-amide functionalized metal-organic framework. *Nat. Commun.* **8,** 14085 doi: 10.1038/ncomms14085 (2017).

**Publisher's note:** Springer Nature remains neutral with regard to jurisdictional claims in published maps and institutional affiliations.

## Supplementary Material

Supplementary Information Supplementary Figures and Supplementary Tables.

Supplementary Data 1 Crystallographic Information File for MFM-188.solv.

Supplementary Data 2 Crystallographic Information File for MFM-188.

Supplementary Data 3 Crystallographic Information File for MFM-188.C_2_D_2_.

Supplementary Data 4 Crystallographic Information File for MFM-188.CO_2_.

## Figures and Tables

**Table 1:** Comparison of CO_2_/C_2_H_2_ uptakes and *Q*_st_ with the density of OMSs and functional groups* for selected MOFs.

**Material**	**BET SA (m**^**2**^** g**^**−1**^**)**	**CO**_**2**_ **uptake**† **(wt%)**	**CO**_**2**_ ***Q***_**st**_ **(kJ mol**^**−1**^**)**	**C**_**2**_**H**_**2**_ **uptake (wt%)**	**C**_**2**_**H**_**2**_ ***Q***_**st**_ **(kJ mol**^**−1**^**)**	**OMS** **density (mmol g**^**−1**^**)**	**FG** **density (mmol g**^**−1**^**)**	**Reference**
MFM-188	2,568	23.7	20.8	27.0‡	32.5	3.3	3.26	This work
NOTT-125	2,471	18.2	25.4	−	−	3.7	3.7	19
Cu_3_(TPBTM)	3,160	23.3	26.3	−	−	3.4	3.4	22
NJU-BAI-17	2,423	−	−	25.8‡	38	4.0	2.0	23
MOF-74Mg	1,495	35.2	47	21.4†	34	8.3	0	27,40
FJI-H8	2,025	−	−	26.0‡	32	3.6	0	11
HKUST-1	1,781	17.9	30.0	23.3‡	30.4	5.0	0	28
ZJU-40	2,858	17.2	24	25.1†	34.5	3.8	3.8	41
SIFSIX-1-Cu	1,178	23.1	27	22.1†	37	0	7.7	29,42
ZJU-8	2,501	20.4	21.9	22.6†	30	3.7	1.8	43
NOTT-101	2,316	−	−	21.34‡	−	3.8	0	44
ZJU-10	2,392	−	−	20.2‡	39	3.7	1.9	45
NOTT-300-Al	1,370	−	−	16.5†	32	0	4.4	7
UTSA-100	970	−	−	11.1‡	22	0	9.7	21
UTSA-60	484	−	−	8.12‡	36	4.2	0	46

FG, functional group; MOFs, metal-organic frameworks; OMS, open metal site; SA, surface area.

^*^Functional groups: MFM-188, amide; NOTT-125, oxamide (considered as two equivalents of amide); [Cu_3_(TPBTM)], amide; NJU-BAI-17, amide; ZJU-40, pyridyl; SIFSIX-1-Cu, fluorine; ZJU-8, amine, ZJU-10, hydroxyl; NOTT-300-Al, hydroxyl; UTSA-100, amine and tetrazolyl.

^†^298 K and 1.0 bar.

^‡^295 K and 1.0 bar.
