# Supplementary material for: Unravelling exceptional acetylene and carbon dioxide adsorption within a tetra-amide functionalized metal-organic framework
Source: Nat Commun. 2017 Feb 8;8:14085. doi: 10.1038/ncomms14085 (PMC5309720; doi:10.1038/ncomms14085)
Supplement: Supplementary Information — Supplementary Figures and Supplementary Tables. [file ncomms14085-s1.pdf]

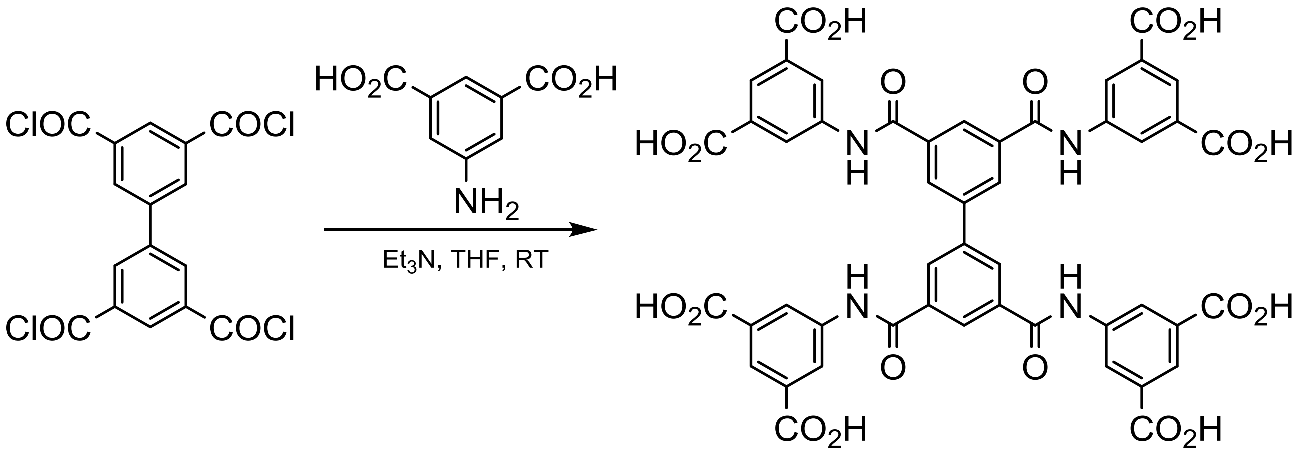

Supplementary Figure 1. Synthetic route to the organic ligand H_8L.

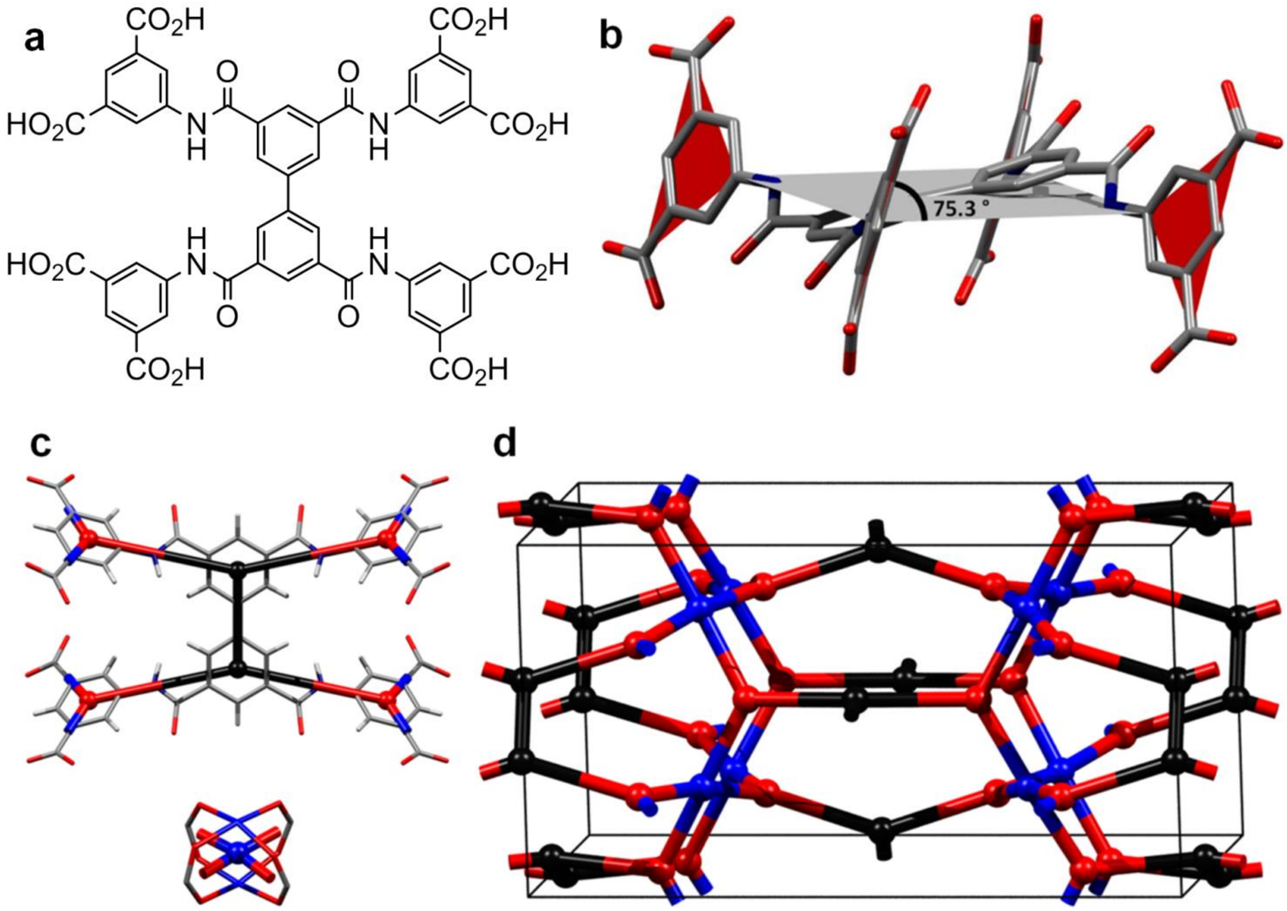

Supplementary Figure 2. Chemical structure of the organic ligand H_8L and the topological analysis of MFM-188. View of the chemical structure of the ligand H_8L (a) and the representation of its conformation in MFM-188 (b). Topological simplifications of the organic ligand and {Cu_2} paddle-wheel (c) and the overall 3,3,4-c ***lwg*** network (d) (4-c nodes from the paddlewheels in blue, 3-c nodes from the isophthalates in red and 3-c nodes from the biphenyl core in black).

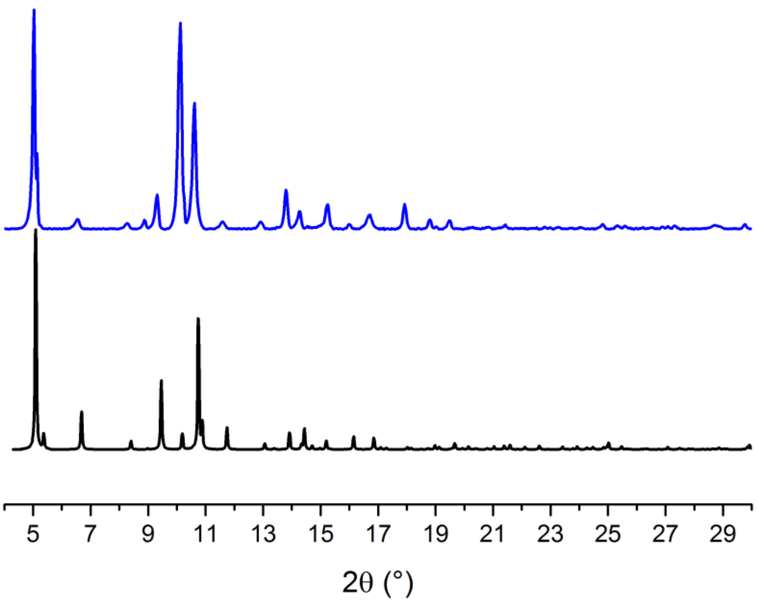

Supplementary Figure 3. Experimental and simulated (from the single crystal structure) PXRD patterns of MFM-188a. The experimental pattern was collected under inert atmosphere using a desolvated sample.

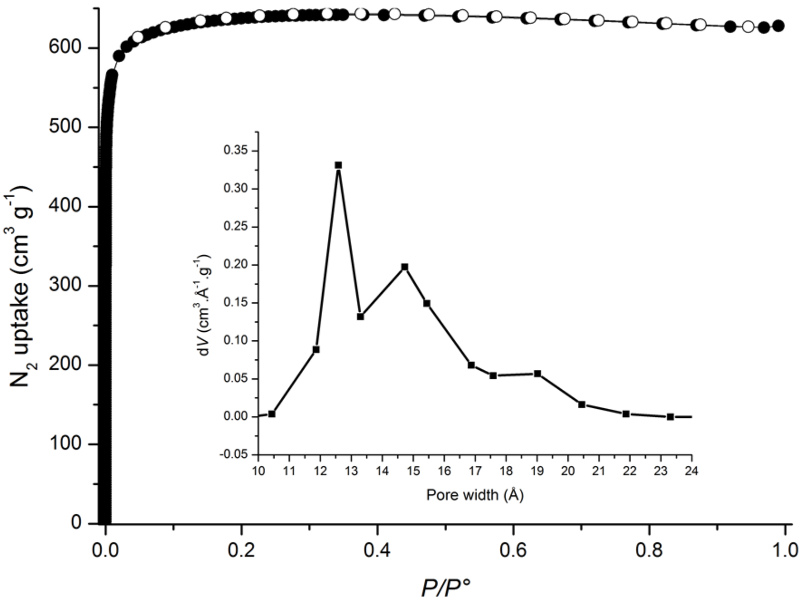

Supplementary Figure 4. N_2 sorption isotherm for MFM-188a at 77 K. Insert plot shows the pore size distribution.

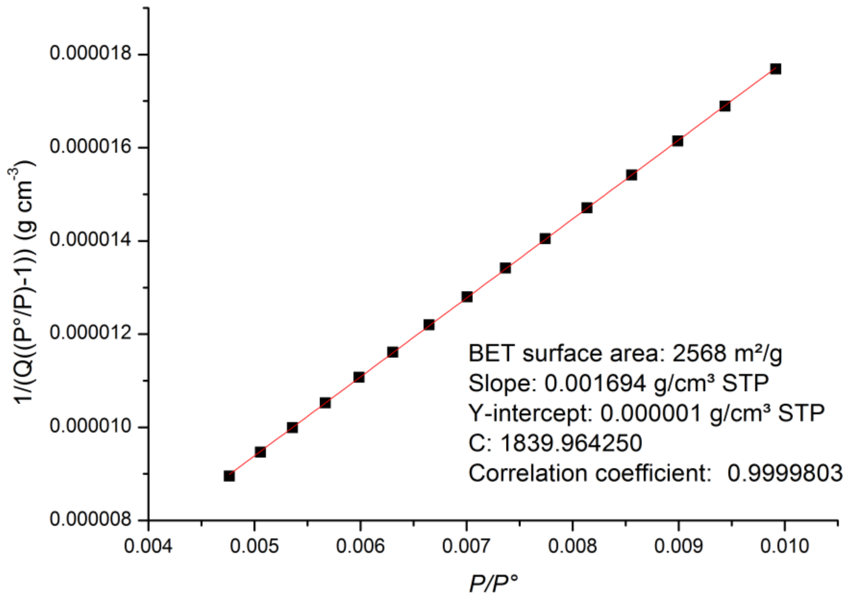

Supplementary Figure 5. The BET plot derived from N_2 uptake of MFM-188a.

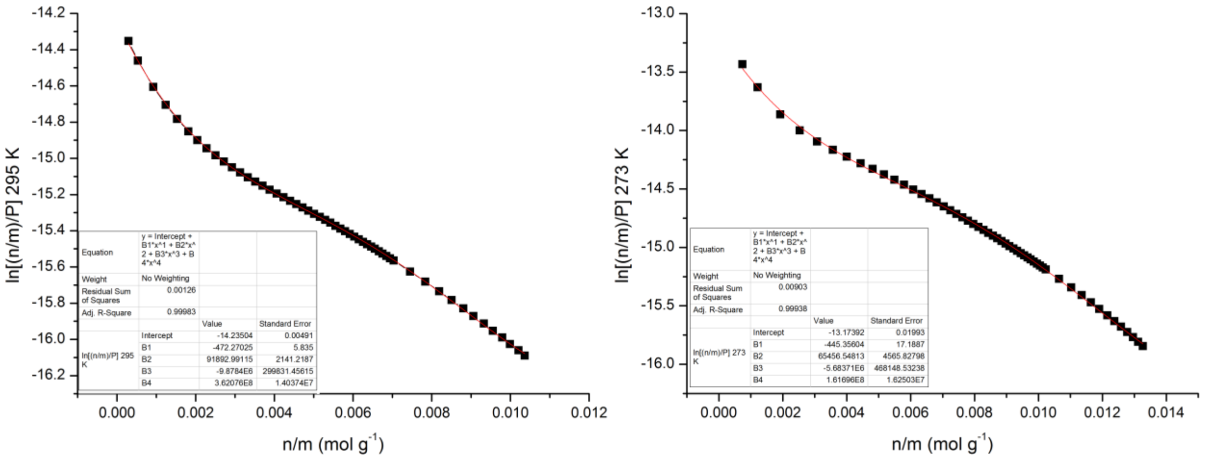

a b

Supplementary Figure 6. Virial fitting of C_2H_2 adsorption isotherm at 295 K (a) and 273 K (b).

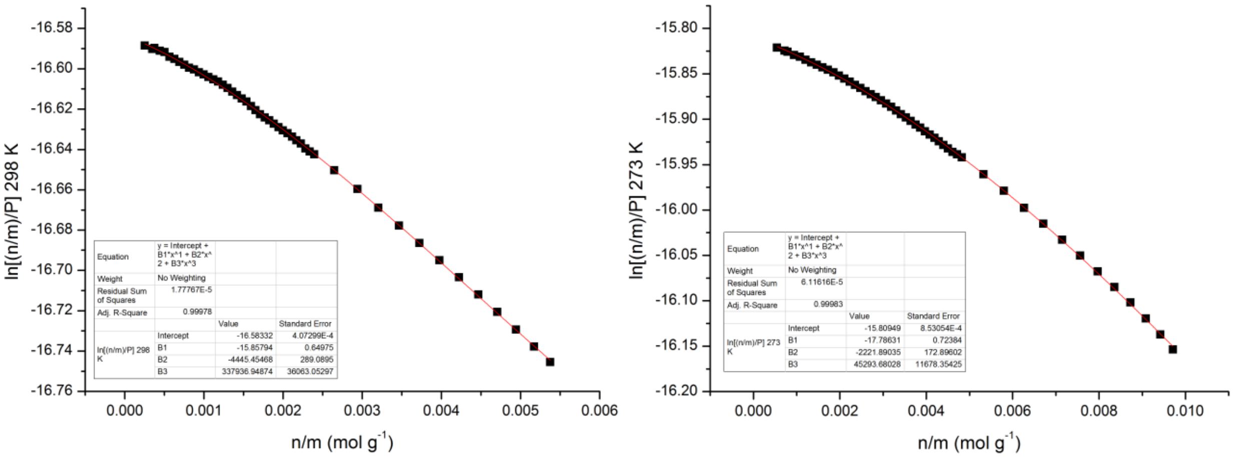

a b

Supplementary Figure 7. Virial fitting of CO_2 adsorption isotherm at 298 K (a) and 273 K (b).

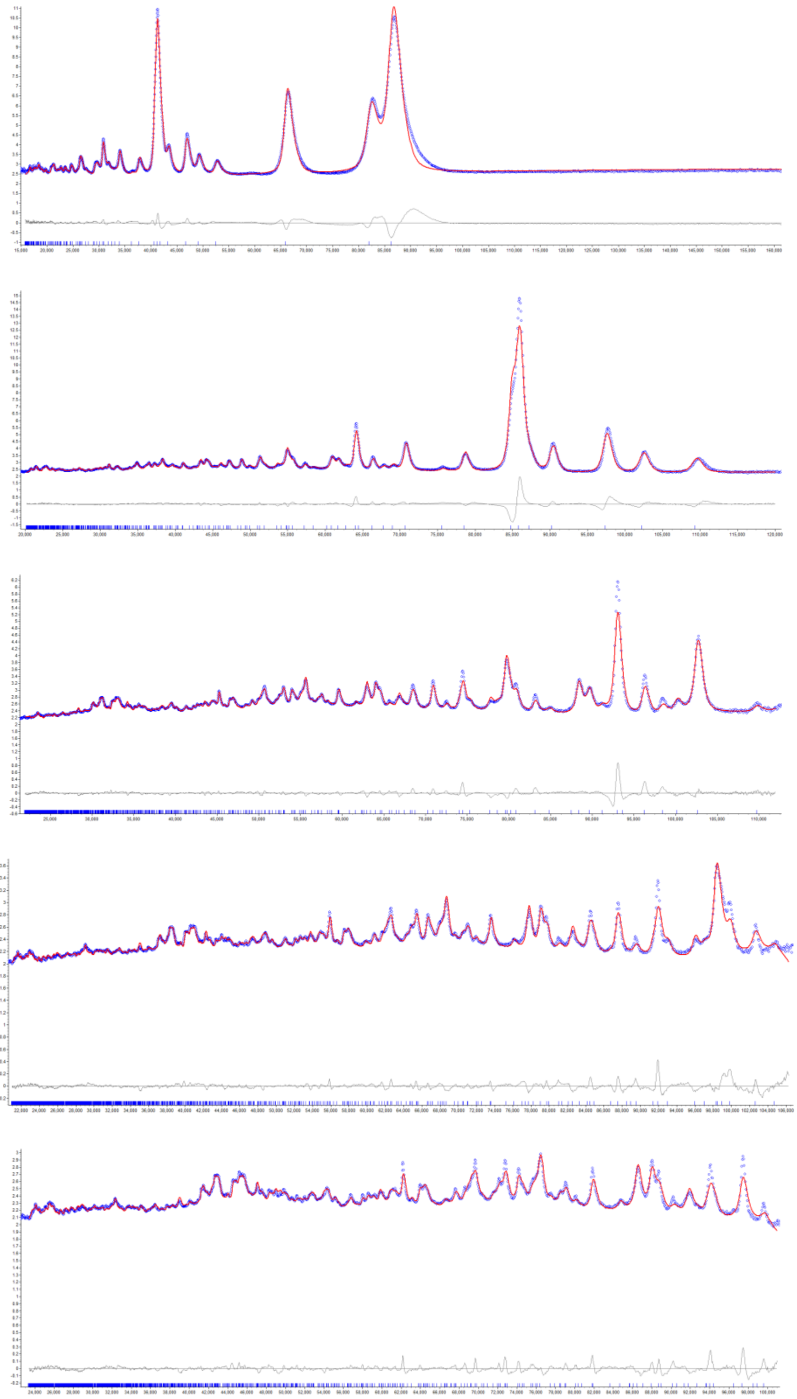

Supplementary Figure 8. Neutron diffraction pattern and Rietveld refinement for desolvated MFM-188a (banks 1 to 5)

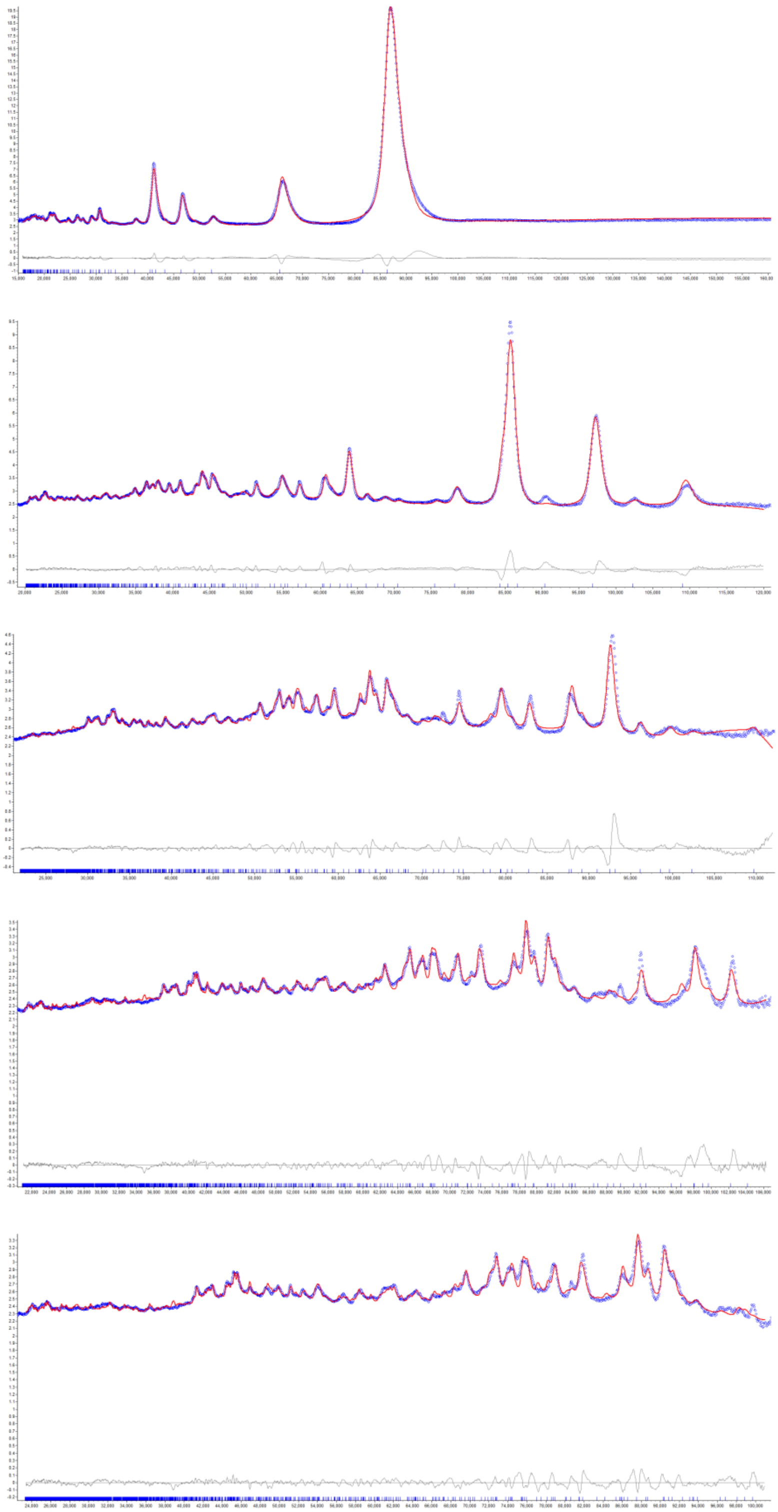

Supplementary Figure 9. Neutron diffraction pattern and Rietveld refinement for 3.157C_2D_2@ MFM-188 (banks 1 to 5)

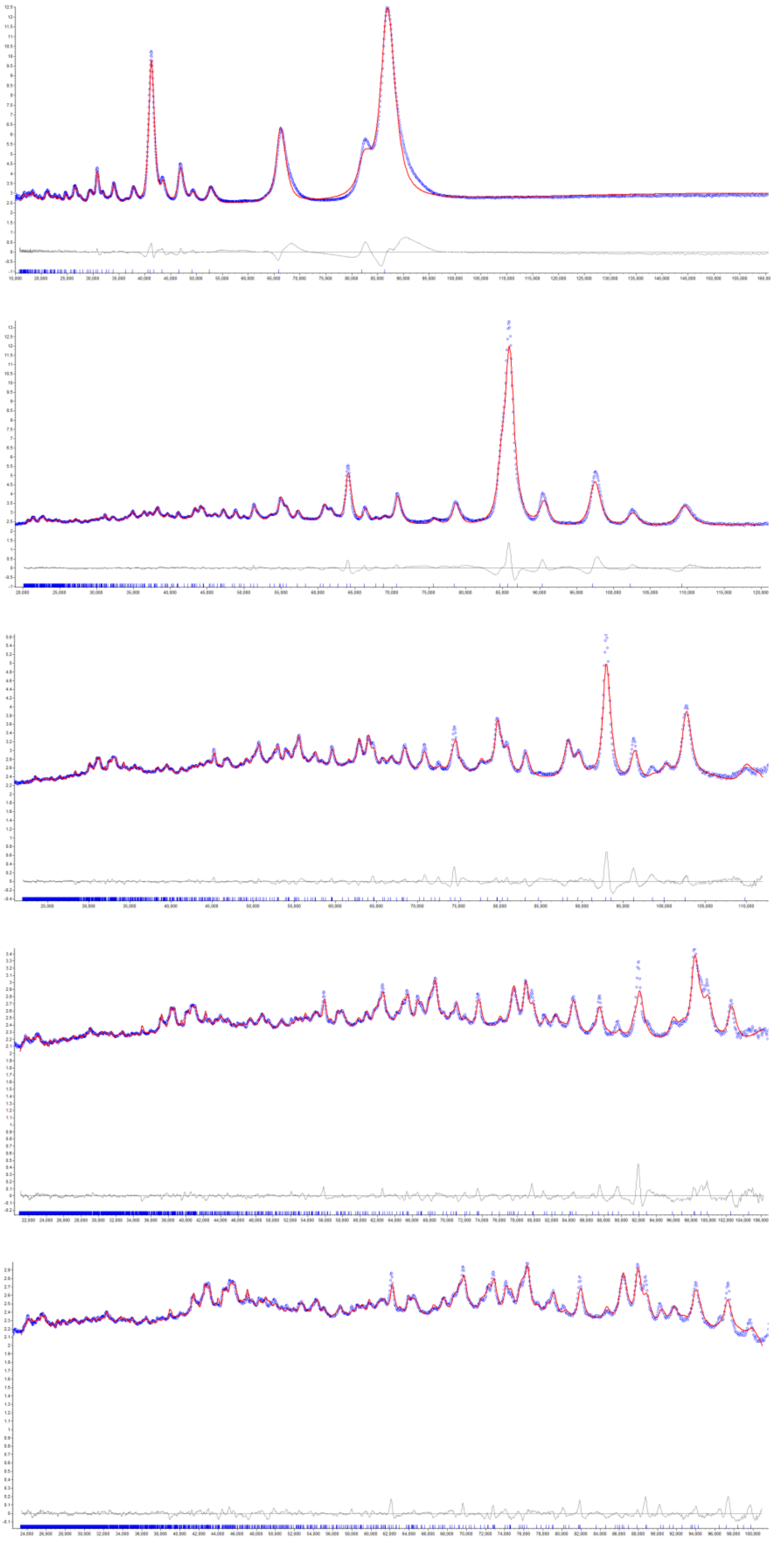

Supplementary Figure 10. Neutron diffraction pattern and Rietveld refinement for 1.75CO_2@ MFM-188 (banks 1 to 5)

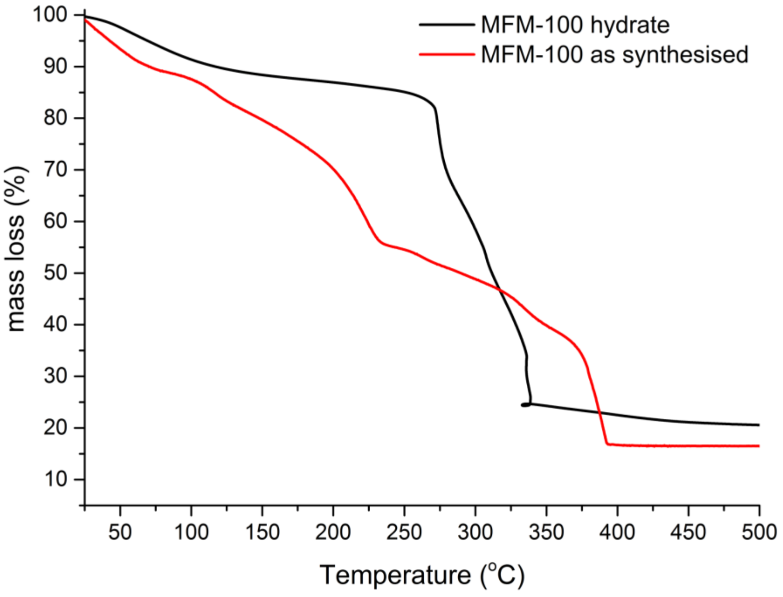

Supplementary Figure 11. TGA plots MFM-188 as synthesized (red) and after desolvation and exposure to ambient conditions (black).

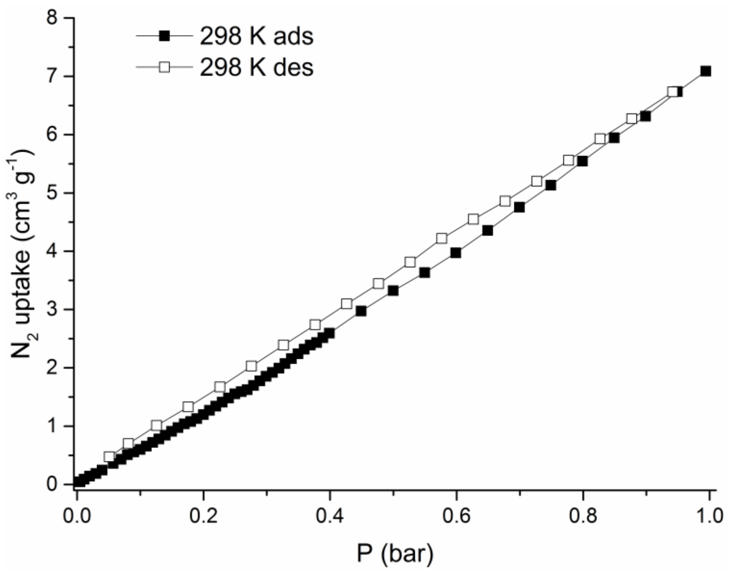

Supplementary Figure 12. N_2 sorption isotherm for MFM-188a at 298 K.

Supplementary Table 1. Single crystal data and structure refinement details for MFM-188.

Identification code	MFM-188
Chemical formula	$(C_{48}H_{30}Cu_4O_{24}N_4)$
M_r (g mol^{-1})	1300.96
Crystal system, space group	Tetragonal, *P4/mnc*
Temperature (K)	120
a, *c* (Å)	18.6841 (7), 34.6796 (17)
V (Å^3)	12106.5 (9)
Z	4
Radiation type	Cu *K*α
μ (mm^{-1})	2.24
Crystal size (mm)	0.14 × 0.14 × 0.01
Absorption correction	Gaussian.
T_{min}, T_{max}	0.747, 0.973
No. of measured, independent and observed [$I > 2\sigma(I)$] reflections	44853, 6006, 3021
R_{int}	0.165
(sin θ/ λ)max (Å-1)	0.625
$R[F^2 > 2\sigma(F^2)]$, $wR(F^2)$, S	0.086, 0.275, 0.92
No. of reflections	6006
No. of parameters	185
H-atom treatment	H-atom parameters constrained
$\Delta\rho_{max}$, $\Delta\rho_{min}$ (e Å^{-3})	1.26, −0.42

Supplementary Table 2. Crystal data and details of the structure determination for C_2D_2@MFM-188 and CO_2@MFM-188.

	Desolvated MFM-188a	**3.157**C_2D_2@MFM-188	**1.75**CO_2@MFM-188
Formula	($C_{48}H_{30}Cu_4O_{24}N_4$)	($C_{48}H_{30}Cu_4O_{24}N_4$) ·3.157($C_2D_2$)	($C_{48}H_{30}Cu_4O_{24}N_4$) ·1.75($CO_2$)
Crystal System	Tetragonal	Tetragonal	Tetragonal
Space group	*P*4/*mnc*	*P*4/*mnc*	*P*4/*mnc*
a, *b* [Å]	18.7721(4)	18.6680(6)	18.7471(5)
c [Å]	34.7704(14)	34.8250(15)	34.8339(18)
V [$Å^3$]	12252.8(7)	12136.3(9)	12242.5(9)
D(calc) [g/cm^3]	0.83341	0.85245	0.83424
Radiation type	Neutron	Neutron	Neutron
Scan method	Time of Flight	Time of Flight	Time of Flight
R_{exp}	0.29	0.28	0.28
R_{wp}	1.87	1.86	1.90
R_p	1.69	1.81	1.77
GoF	6.49	6.63	6.72
